# Topobexin Targets a Unique Druggable Pocket of Topoisomerase II for Beta Isoform- Selective Control of DNA Damage During Anthracycline Chemotherapy

**DOI:** 10.1063/4.0001148

**Published:** 2025-10-27

**Authors:** Jan Kubes, Galina Karabanovich, Anh T. Q. Cong, Iuliia Melnikova, Olga Lencova, Petra Kollarova, Hana Bavlovic Piskackova, Veronika Kerestes, Lenka Applova, Lise C. M. Arrouye, Julia R. Alvey, Jasmina Paluncic, Taylor L. Witter, Anna Jirkovska, Jiri Kunes, Petra Sterbova-Kovarikova, Caroline A. Austin, Martin Sterba, Tomas Simunek, Jaroslav Roh, Matthew J. Schellenberg

**Affiliations:** 1 Charles University, Hradec Kralove, HK, Czech Republic; 2 Mayo Clinic, Rochester, MN, USA; 3 Charles University, Rochester, HK, Czech Republic; 4 Newcasle University, Newcastle Upon Tyne, UK, United Kingdom

## Abstract

Anthracyclines are potent chemotherapeutic agents, yet their clinical use is hampered by their cardiotoxicity. Anthracyclines achieve potent anti- cancer effects primarily via TOP2A, but at the same time they induce a dose limiting cardiotoxicity through TOP2B. The use of a catalytic TOP2 inhibitors such as dexrazoxane (ICRF 187) significantly reduces the incidence of anthracycline-induced cardiotoxicity. However, dexrazoxane targets the highly conserved TOP2 dimer interface that is identical between TOP2A and TOP2B, fostering persisting concerns that its inhibition of TOP2A isoenzyme may interfere with anthracycline anticancer effects and augment anthracycline-induced myelosuppression. Thus, isoform- selective TOP2 inhibitors that can control the DNA damage caused by TOP2A vs TOP2B are a significant unmet clinical need.

We have developed a new class of highly potent and TOP2B-selective catalytic inhibitors through rational design by building upon the general pharmacophore present in isolates from Calophyllum Brasiliense. Using X-ray crystallography, we demonstrate that our newly developed compounds occupy a previously unidentified druggable binding-site in the TOP2 ATPase domain and function as a physical barrier to block the conformational change triggered by ATP hydrolysis. We have named the class of inhibitors that target this site “obex”, the Latin word for obstacle. The obex binding site contains residues that differ between the TOP2A and TOP2B isoenzymes, based on which we rationally designed, prepared, and systematically evaluated a targeted panel of obex inhibitors. We have identified the lead molecule, namely, topobexin, as the first- in-class TOP2B-specific obex inhibitor that can prevent anthracycline-induced DNA damage. This first TOP2 isoform-specific inhibition underscores the broader potential to improve drug specificity and minimize adverse effects in various medical treatments.

